# SOD mediates mitochondrial epigenetic regulation in NIHL

**DOI:** 10.3389/fncel.2025.1673070

**Published:** 2025-10-15

**Authors:** Liuwei Shi, Caiping Li, Dianpeng Wang, Dafeng Lin, Xiangli Yang, Peimao Li, Wen Zhang, Yan Guo, Liting Zhou, Naixing Zhang

**Affiliations:** 1 Medical Laboratory, Shenzhen Prevention and Treatment Center for Occupational Diseases, Shenzhen, China; 2 School of Public Health, Jilin University, Changchun, China; 3 Department of Toxicology, School of Public Health, Southern Medical University, Guangzhou, China

**Keywords:** occupational noise-induced hearing loss, mitochondrial DNA, D-loop methylation, oxidative stress, superoxide dismutase (SOD), epigenetic regulation

## Abstract

Occupational noise-induced hearing loss (NIHL) is linked to the overproduction of mitochondrial reactive oxygen species after noise exposure. This cross-sectional study investigated the relationship between mitochondrial DNA (mtDNA) D-loop region methylation and oxidative stress in 150 participants divided into three age and sex matched groups: a control group (*n* = 50, workers without noise exposure and with normal hearing), an exposed group (*n* = 50, workers with significant noise exposure but normal hearing), and a case group (*n* = 50, workers diagnosed with NIHL). The subjects among groups were matched for sex and age to control confounding factors. Methylation levels of the mtDNA D-loop region were determined by the quantitative PCR following bisulfite conversion, while mitochondrial DNA copy number (mtDNA-CN) was assessed using the real-time PCR. Oxidative stress markers—including superoxide dismutase (SOD), glutathione peroxidase (GPX), total antioxidant status (TAS), and malondialdehyde (MDA)—were quantified via substrate-specific assays, ultraviolet enzymatic methods, and colorimetric techniques. Results showed the case group (141.6 ± 46.80 U/mL) showed lower SOD than the control (159.5 ± 18.68 U/mL, *p* < 0.05) and exposed groups (164.0 ± 15.44 U/mL, *p* < 0.01), MDA was higher in the case group (232.8 ± 134.5 nmol/mL) than in the control (193.5 ± 84.13 nmol/mL) and exposed groups (187.3 ± 60.76 nmol/mL), with a significant overall difference (*F* = 3.162, *p* < 0.05). The case group showed lower methylation [1.205 (0.595, 2.748) %] than both the control [1.710 (0.912, 3.225) %] and exposed groups [1.850 (0.987, 4.093) %] (H = 7.492, *p* < 0.05). The case group exhibited higher mtDNA-CN levels [397.7 (205.9, 532.1)] compared to both the blank control group [317.4 (234.6, 549.6)] and the exposed group [225.1 (125.3, 445.0)] (H = 9.213, *p* < 0.05). Methylation levels of the D-loop region were positively correlated with SOD and negatively correlated with MDA. Mediation analysis indicated that SOD may mediate the relationship between D-loop methylation and bilateral high-frequency hearing thresholds, suggesting an indirect epigenetic regulatory mechanism. These findings imply that noise-induced oxidative imbalance, reflected by reduced SOD, may lead to D-loop hypomethylation, contributing to the development of NIHL. These methylation sites may serve as preliminary biomarkers for further research on preventive strategies.

## 1 Introduction

NIHL is an auditory impairment resulting from exposure to high sound levels, whether from a single intense burst or prolonged exposure (Lie et al., 2016). It represents one of the most prevalent occupational hazards worldwide. Epidemiological studies indicate significant regional variation, with prevalence rates of 25% in the United States, 15% in Canada, and approximately 20% across the European Union and Australia (Themann and Masterson, 2019; Teixeira et al., 2021), with rising rates in developing countries (Fuente and Hickson, 2011). In China, NIHL prevalence exceeds 20%, making it the second most common occupational disease, and the incidence continues to rise (Zhou et al., 2020). Beyond auditory damage, noise exposure is linked to various psychological and physiological effects, including tinnitus, cardiovascular dysfunction, cognitive impairment, and sleep disturbances (Ellermeier et al., 2001; Mohammad et al., 2023). The mechanism of cochlear damage in NIHL is largely mediated by reactive oxygen species (ROS) generated in the inner ear following acoustic overexposure (Zhou et al., 2023). Animal models have demonstrated that noise triggers apoptotic pathways leading to cochlear cell death, with ROS levels peaking within 2 weeks post-exposure (Kishimoto-Urata et al., 2022). Initial hair cell damage is attributed to mechanical trauma and acute ROS overload (Mao and Chen, 2021), while sustained ROS and reactive nitrogen species (RNS) production contribute to progressive cell loss (Kamogashira et al., 2015). In guinea pig models, noise exposure induces the mitochondrial release of apoptosis-inducing factors, concomitant with reduced ATP synthesis and elevated ROS, activating apoptosis and resulting in outer hair cell death (Rommelspacher et al., 2024).

Mitochondrial dysfunction plays a central role in NIHL pathology. Noise exposure enhances mitochondrial aerobic respiration, increasing ROS production and promoting inner ear hair cell apoptosis (Samara et al., 2024). The antioxidant system—including enzymes such as SOD, GPX and TAS—partially counteracts ROS effects, underscoring the significance of mitochondrial impairment in NIHL development.

DNA methylation, an essential epigenetic mechanism involving the addition of methyl groups to cytosine bases, regulates gene expression without altering the DNA sequence (Dhar et al., 2021). It is modulated by various environmental factors (Venney et al., 2021). Although nuclear DNA methylation has been extensively studied, research on mitochondrial DNA (mtDNA) methylation remains limited. Recent evidence highlights the functional importance of mtDNA methylation in pathological and physiological processes, including deafness (Donato et al., 2024).

Mitochondria, which are central to oxidative phosphorylation and apoptosis, possess inadequate DNA repair mechanisms, rendering mtDNA highly vulnerable to oxidative damage (Khan et al., 2022; Sergeeva et al., 2023; Flores et al., 2013; Wong and Ryan, 2015). The D-loop region of mtDNA regulates mitochondrial gene transcription and function, (Lei et al., 2024) and its aberrant methylation is linked to mitochondrial dysfunction and oxidative stress in neurological disorders (Trinchese et al., 2024).

Epigenetic regulation, particularly DNA methylation, is increasingly associated with hearing loss. Studies have identified methylation differences at CpG sites related to various hearing loss phenotypes (Zheng et al., 2021; Rizk et al., 2022). Genome-wide methylation analyses have correlated epigenetic changes with auditory function, implicating genes such as TCF25 and FGDR1 (Guo et al., 2023). Environmental exposures, such as lead from electronic waste, can also induce epigenetic modifications affecting auditory development (Xu et al., 2020). Such findings suggest that mtDNA D-loop methylation could be a key target for exploring NIHL pathogenesis (Basu et al., 2020). Moreover, mtDNA copy number (mtDNA-CN), a biomarker of mitochondrial integrity, fluctuates in response to damage and oxidative stress (Castellani et al., 2020). Both human and animal studies have shown that noise exposure alters mtDNA-CN (Yang et al., 2024; Yu et al., 2014; Tan and Song, 2023). Despite advances, research on mitochondrial epigenetic mechanisms in NIHL remains limited, especially concerning the interplay between oxidative stress and mtDNA methylation. This study aims to investigate changes in mtDNA D-loop region methylation and oxidative stress profiles in individuals with occupational NIHL, to elucidate potential epigenetic regulatory pathways involved in its pathology.

## 2 Methods

### 2.1 Cases and controls

This study recruited a total of 150 participants and divided them into three groups. Within the population engaged in noisy occupations, 50 confirmed cases of noise-induced hearing loss were selected to constitute the case group. Additionally, 50 noise-exposed workers with normal hearing, as determined by their occupational health examinations, were selected to form the exposed group. A control group was composed of 50 employees with normal hearing who had never employed hearing examinations for noise-related occupations. All groups of study subjects were matched for gender and age (±5 years). Inclusion criteria: ① Case and exposed groups: subjects with occupational noise exposure for ≥3 years; control group: subjects without occupational noise exposure. ② According to GBZ49-2014 “Diagnosis of Occupational Noise-Induced Hearing Loss,” the case group was diagnosed with occupational NIHL, defined as a bilateral high-frequency (3,000 Hz, 4,000 Hz, 6,000 Hz) average hearing threshold of ≥40 dB. For the control group and exposed group, normal hearing was defined as a high-frequency average threshold of <35 dB and a pure tone audiometry threshold of ≤25 dB at any frequency range (500 Hz, 1,000 Hz, and 2000 Hz) in either ear. Exclusion criteria: Participants were excluded if they had any of the following conditions: pseudohypacusis, exaggerated hearing loss, drug-induced ototoxicity (e.g., Streptomycin, Kanamycin, Chloramphenicol), traumatic hearing loss, infectious diseases (such as epidemic cerebrospinal meningitis, mumps, and measles), hereditary deafness, Meniere’s disease, sudden deafness, middle ear diseases, acoustic neuroma, or auditory nerve diseases. A 7.0 mL sample of upper limb venous blood should be collected from each experimental subject and aliquoted into two types of tubes: (1) regular biochemical tubes (without anticoagulant) and (2) EDTA-anticoagulant tubes. Two milliliters of peripheral blood were collected in EDTA anticoagulant tubes for use as the detection sample, and routine blood tests were conducted using a hematology analyzer (Mindray BC5000, Shenzhen China). Data on the study subjects’ age, blood pressure, occupational exposure duration, and bilateral high-frequency average threshold were retrieved from the Electronic Health Record System. The study cohort comprised both confirmed patients and healthy individuals who underwent medical examinations at the Shenzhen Occupational Disease Prevention and Treatment Center. This study was approved by the Ethics Committee of the Shenzhen Occupational Disease Prevention and Treatment Center, and all study subjects provided informed consent.

### 2.2 Mitochondrial DNA D-loop methylation level determination

The total DNA was extracted by DNA extraction kit by Shanghai Bioengineering Co, Ltd. A 400 μL aliquot of whole blood was transferred to a 1.5-mL microcentrifuge tube. Subsequently, 40 μL of Proteinase K was added and mixed thoroughly by vortexing. Then, 400 μL of Buffer DL was added, and the mixture was inverted gently to homogenize. The solution was incubated in a 56 °C water bath for 10 min. Following incubation, 400 μL of absolute ethanol was added to the tube, and the mixture was inverted vigorously to ensure complete precipitation of nucleic acids. The lysate (650 μL) containing suspended particles was carefully pipetted into a pre-assembled adsorption column placed in a collection tube. After standing for 2 min, the column was centrifuged at 10,000 rpm for 1 min at room temperature. The flow-through was discarded, and this step was repeated until all lysate had passed through the column. 500 μL of GW Solution was added, followed by centrifugation at 10,000 rpm for 30 s. 700 μL of Wash Solution was applied, and the column was centrifuged again under identical conditions. A final wash with 500 μL of GW Solution was conducted, followed by centrifugation. All wash effluents were discarded after each step. To eliminate residual ethanol, the column was centrifuged at 12,000 rpm for 2 min.

The dried column was transferred to a new 1.5-mL microcentrifuge tube. For optimal DNA recovery, 15 μL of CE Buffer (pre-heated to 60 °C for 10 min) was added directly to the center of the column membrane. After a 3-min incubation at room temperature, the DNA was eluted by centrifugation at 12,000 rpm for 2 min. This elution step was repeated once with an additional 30 μL of CE Buffer. The purified DNA was either used immediately for downstream applications or stored at −20 °C for long-term preservation. Bisulfite conversion of genomic DNA (gDNA) was performed using the Zymo EZ DNA Methylation Lightning MagPrep Kit (Catalog No. D5046).

The design and synthesis of primers are based on the retrieval of the human DNA sequence of the target gene D-loop from the NCBI database (Gene ID: NC_012920), The *β* - actin internal reference gene sequence was sourced from the reference literature (Hulbert et al., 2017). Design the primers using the MethPrimer website, and have them synthesized by Shanghai Biotech Co., Ltd. (Table 1).

**Table 1:** Primer and probe sequences.

Gene	Primer	Sequence(5′ - 3′)
MET D-LOOP	Forward	GGTTTATTATTTTATTAATTATTTACGG
	Reverse	ATAAAATACTCCGACTCCAACGTC
	probe	FAM-TTTTTTATGTATTTGGTATTTT-MGB
β-ACTIN	Forward	TGGTGATGGAGGAGGTTTAGTAAGT
	Reverse	AACCAATAAAACCTACTCCTCCCTTAA
	probe	FAM-ACCACCACCCAACACACAATAACAAACACA-TAMRA

The quantitative PCR (qPCR) reaction system and conditions were established following the guidelines provided by the SuperReal Color Fluorescent Quantitative Pre-Mix Kit. The reagents included in the kit were thoroughly mixed by inversion and subsequently centrifuged immediately. Amplifcation of the two reactions using primer Forward and primer Reverse of 0.5 each μL (pmol/μL). Probe 0.6 μL (pmol/μL). Water 4.4 μL. SuperReal Permix 10 μL. DNA template 4 μL. 20 in total μL (Table 1). The reaction system was assembled on ice and subsequently aliquoted into eight tubes devoid of RNA enzymes for machine detection. Each sample was analyzed in triplicate wells. The proportion of DNA methylation was calculated using the CT difference method based on literature (Park et al., 2021). The PCR amplification parameters for the D-loop and *β*-actin gene were established as follows: an initial denaturation at 95 °C for 15 min, followed by 45 cycles of denaturation at 95 °C for 3 s, and annealing/extension at 57 °C for 45 s. Mitochondrial DNA copy number detection samples are derived from peripheral blood samples. For this study, hemoglobin, beta gene and Mitochondrial DNA Copy Number Analysis.

To quantify mitochondrial DNA (mtDNA) copy number, the hemoglobin beta gene (HBB; GenBank: MH708880.1) and mitochondrial NADH dehydrogenase 1 gene (MT-ND1; GenBank: NC_012920.1) were selected as nuclear and mitochondrial reference genes, respectively, due to their stable expression. A total of 20 ng of cellular DNA was used as template in a 20 μL reaction mixture containing 10 μL of 2 × SYBR Green Master Mix (Tiangen, Cat. No. FP215), 10 pmol of each primer, and 2 μL DNA. Reactions were performed in triplicate on a StepOnePlus Real-Time PCR System (Applied Biosystems) under the following conditions: initial denaturation at 95 °C for 10 s; 40 cycles of denaturation at 95 °C for 5 s, and annealing/extension at 56 °C for 34 s. HBB (nuclear reference): Forward: 5′-GCTTCTGACACAACTGTGTTCACTAGC-3′, Reverse: 5′-CACCAACTTCATCCACGTTCACC-3′. MT-ND1 (mitochondrial target, chrM:3313–3322): Forward: 5′-CACCCAAGAACAGGGTTTGT-3′, Reverse: 5′-TGGCCATGGGTATGTTGTTA-3′. Threshold cycle (Ct) values were determined using Applied Biosystems software. The relative mtDNA -CN was calculated using the ΔCt method, expressed as the ratio of MT-ND1 (mitochondrial) to HBB (nuclear) signals (Wang et al., 2024).

### 2.3 The determination of the oxidative stress index included SOD, GAX, and TAS

Five milliliters of peripheral blood were centrifuged at 2,000 g for 30 min to isolate serum for subsequent analyses. SOD activity was quantified using the substrate method. Under alkaline conditions, pyrogallol undergoes autoxidation to form purpurogallin and superoxide anion (O₂^−^). The rate of pyrogallol autoxidation is correlated with the concentration of O₂^−^. SOD catalyzes the dismutation of O₂^−^ into hydrogen peroxide (H₂O₂) and oxygen (O₂), thereby inhibiting the autoxidation of pyrogallol. By measuring the absorbance changes of purpurogallin at a wavelength of 405 nm, the SOD activity in the sample can be determined. GPX activity was measured via the UV enzyme assay. GPX catalyzes the oxidation of reduced glutathione (GSH) to oxidized glutathione (GSSG) using cumene hydroperoxide as a substrate. In the presence of glutathione reductase (GR) and reduced nicotinamide adenine dinucleotide phosphate (NADPH), GSSG is rapidly reduced back to GSH, while NADPH is oxidized to nicotinamide adenine dinucleotide phosphate (NADP⁺). The rate of NADPH oxidation is directly proportional to the activity of GPX in the serum. By measuring the rate of decrease in NADPH absorbance at 340 nm, the activity of GPX can be determined. TAS levels were determined using the colorimetric method. In the presence of an oxidizing agent, 2,2′-azino-bis (3-ethylbenzothiazoline-6-sulfonic acid) diammonium salt (ABTS) is oxidized to generate the ABTS⁺· radical cation, which exhibits a stable bluish-green color and can be measured at 600 nm. Antioxidants can scavenge these radicals, inhibiting the formation of the colored product and resulting in a decrease in absorbance. The degree of inhibition is proportional to the concentration of the antioxidant, thereby allowing for the assessment of the sample’s antioxidant capacity. MDA levels were determined using the colorimetric method. Under acidic conditions, malondialdehyde (MDA) reacts with thiobarbituric acid (TBA) to form a red-colored product, which exhibits a maximum absorption peak at 532 nm. By measuring the absorbance at 532 nm, the concentration of MDA in the sample can be determined. The SOD, GPX, and TAS assay kits were obtained from Zhongtuo Biological Co., Ltd. (China). The MDA assay kit was obtained from the Nanjing Jiancheng Bioengineering Institute (China).

### 2.4 The mediation effect was analyzed using SPSS software

To explore the intrinsic mechanism of the significant positive impact of methylation on bilateral high-frequency hearing thresholds, SOD is further introduced as a mediator variable in the study. Age, diastolic pressure, systolic pressure, TAS, and mitochondrial copy number are controlled variables in the structural equation model.

### 2.5 Statistical analysis

Statistical analysis of all data was performed using IBM SPSS 24.0 software. The data for each group were represented as x̅±s or M (P25, P75). For normally distributed and homogenous data, t-test was used for comparison between two groups, one-way analysis of variance (ANOVA) was used for comparison among multiple groups, and Tukey’s test was used for pairwise comparisons between groups. For non-normally distributed or heteroscedastic data, Kruskal-Wallis test was used for comparison among groups. A significance level of *p <* 0.05 was considered statistically significant. Through using Model4 in the SPSS macro program Process to test the mediating effect, analysis verification is conducted based on the Bootstrap method provided by Hayes. Enrichment analysis of functions and signaling pathways associated with MT genetic loci exhibiting differential methylation expression was conducted using KOBAS v3.0^1^ based on the KEGG database, applying a corrected *p*-value threshold of < 0.05.

## 3 Result

### 3.1 General characteristics

Given the predominance of male patients with noise-induced hearing loss, the study participants in this experiment were exclusively male. As indicated in Table 2, there was no statistically significant difference in age among the three groups of subjects (*F* = 2.07, *p =* 0.13). However, the systolic blood pressure in the case group was significantly higher than that in both the control group and the exposed group, with the differences among the three groups reaching statistical significance (*F* = 5.23, *p <* 0.01). The diastolic blood pressure in the case group was significantly elevated compared to both the control group and the exposed group, with the differences among the three groups reaching statistical significance (*F* = 3.78, *p <* 0.05). Additionally, the hearing threshold in the case group was markedly higher than that in the control group and the exposed group, with the differences among the three groups demonstrating high statistical significance (*F* = 559.5, *p <* 0.001). The absolute lymphocyte count in the case group was lower compared to both the control group and the exposed group; however, the differences among the three groups were not statistically significant (H = 0.86, *p =* 0.651). Similarly, the platelet-to-lymphocyte ratio (PLR) in the case group was higher than that in the control group and the exposed group, yet the differences were also not statistically significant (H = 0.9, *p =* 0.64) (Table 2).

**Table 2:** The basic characteristics of the three study groups.

Characteristic	Control *n* = 50	Exposed *n* = 50	Case *n* = 50	*P*
Age (year)	42.42 ± 6.312	44.80 ± 7.461	45.00 ± 7.321	0.13^a^
Systolic pressure (mm Hg)	119.7 ± 12.15	120.8 ± 11.97	127.3 ± 14.68	0.006^a^
Diastolic pressure (mm Hg)	77.62 ± 8.557	80.18 ± 9.413	82.76 ± 10.01	0.025^a^
Binaural average hearing threshold (dB)	6.710 ± 5.701	6.206 ± 4.913	58.06 ± 13.47	<0.001^a^
absolute lymphocyte count (×10^9^/L)	2.07 (1.633, 2.463)	2.15 (1.598, 2.608)	1.97 (1.65, 2.33)	0.651^b^
Platelet-to-lymphocyte ratio (PLR)	119.9 (99.58, 138.5)	107.1 (93.81, 151.9)	121.9 (99.55, 137.1)	0.637^b^

a One way ANOVA.

b Kruskal-Wallis test.

### 3.2 Comparison of oxidative stress levels in the subjects

**Figure 1:**
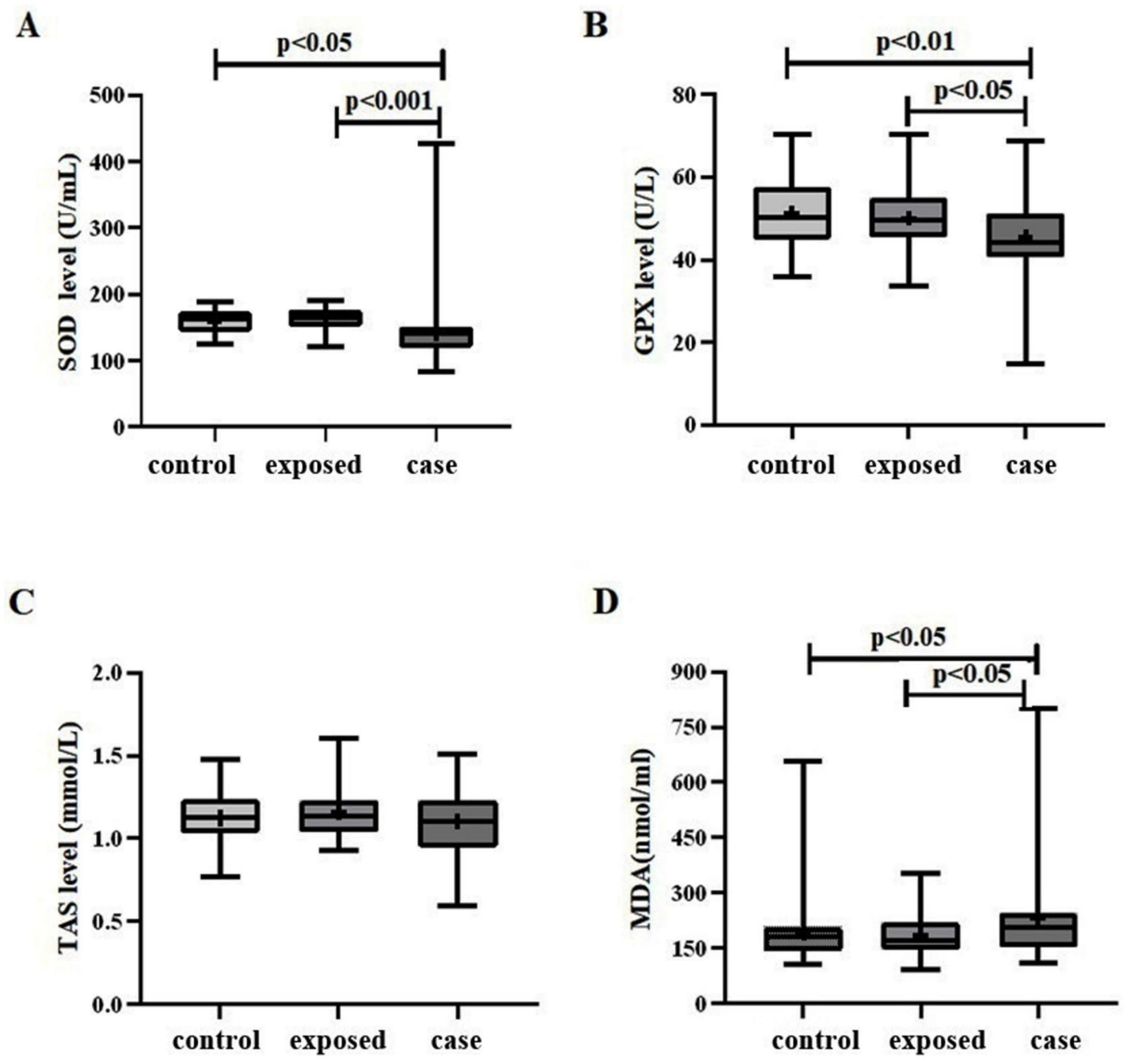
Oxidative stress levels in three study groups. **(A)** A significant difference in SOD levels was observed among the three groups (*F* = 7.599, *p* < 0.01). Specifically, the case group (141.6 ± 46.80) exhibited lower SOD levels compared to both the control group (159.5 ± 18.68), *p* < 0.05, and the exposed group (164.0 ± 15.44), *p* < 0.01. **(B)** Similarly, a significant difference in GPX levels was found among the three groups (*F* = 5.631, p < 0.01). The case group (45.66 ± 10.61) demonstrated lower GPX levels compared to the control group (51.36 ± 8.286), *p* < 0.01, and the exposed group (50.13 ± 7.651), *p* < 0.05. **(C)** The TAS level in the case group (1.105 ± 0.1923) was lower than that in both the control group (1.125 ± 0.1491) and the exposed group (1.163 ± 0.1688); however, this difference was not statistically significant (*F* = 1.514, *p* = 0.224). **(D)** The MDA level in the case group (232.8 ± 134.5) was higher than that in the control group (193.5 ± 84.13) and the exposed group (187.3 ± 60.76), with a statistically significant difference observed among the three groups (*F* = 3.162, *p* < 0.05).

The levels of antioxidant indicators SOD, GPX and TAS were significantly reduced in the case group relative to both the control group and the exposed group, with the exposed group exhibiting the highest levels among the three groups (Figure 1). Conversely, the concentration of MDA, an indicator of oxidative damage, was elevated in the case group compared to the control group and the exposed group, with the control group demonstrating the lowest levels among the three groups.

#### 3.2.1 Comparison of methylation levels in the D-loop region among three subject groups

**Figure 2:**
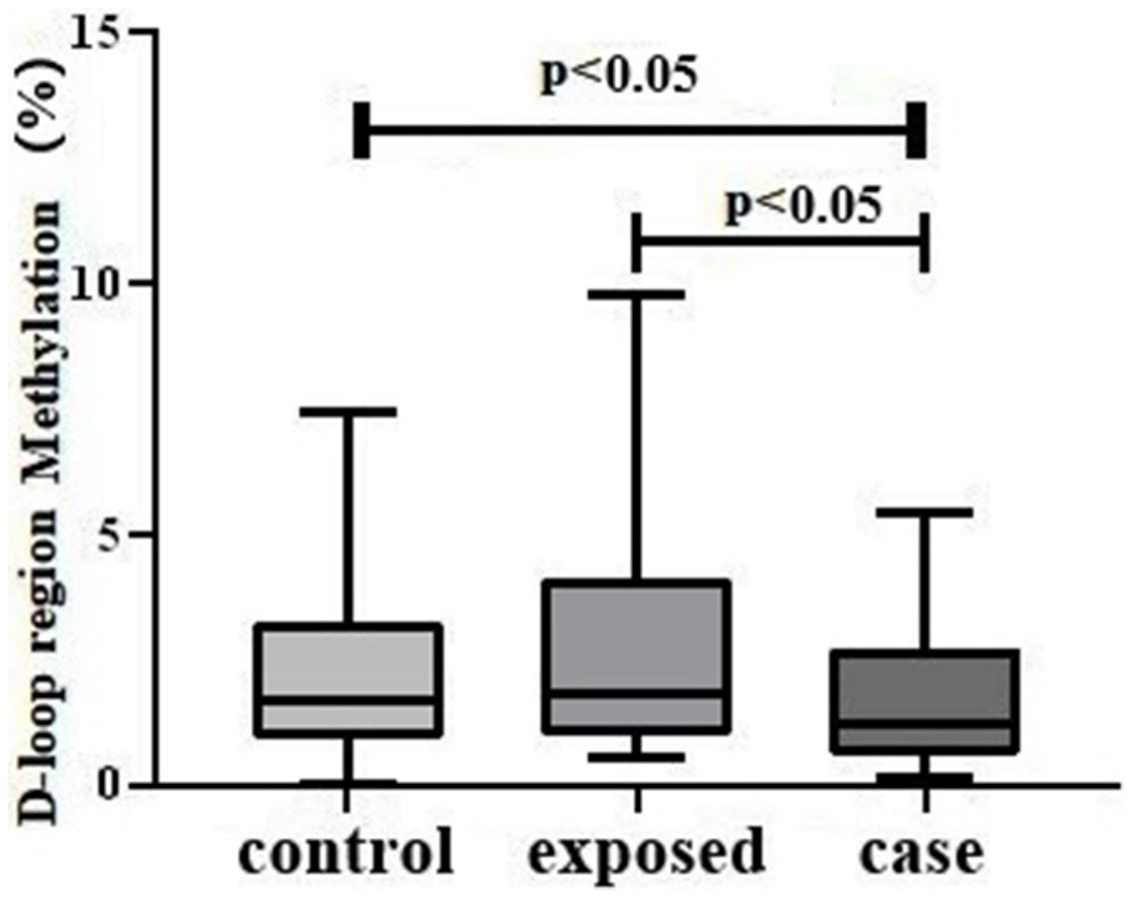
D-loop methylation Levels in Three Study Groups. The results indicate a statistically significant difference in mtDNA D-loop methylation across the groups (H = 7.492, *p* < 0.05). Specifically, the case group exhibited lower methylation levels [1.205 (0.5950, 2.748)] compared to both the control group [1.710 (0.9125, 3.225)] and the exposed group [1.850 (0.9875, 4.093)], with *p*-values less than 0.05.

The levels of mtDNA D-loop methylation were significantly reduced in the case group relative to both the control group and the exposed group, with the exposed group exhibiting the highest levels among the three groups (Figure 2).

### 3.3 The correlation between DNA methylation levels and average hearing threshold of high-frequency bilateral ears

**Figure 3:**
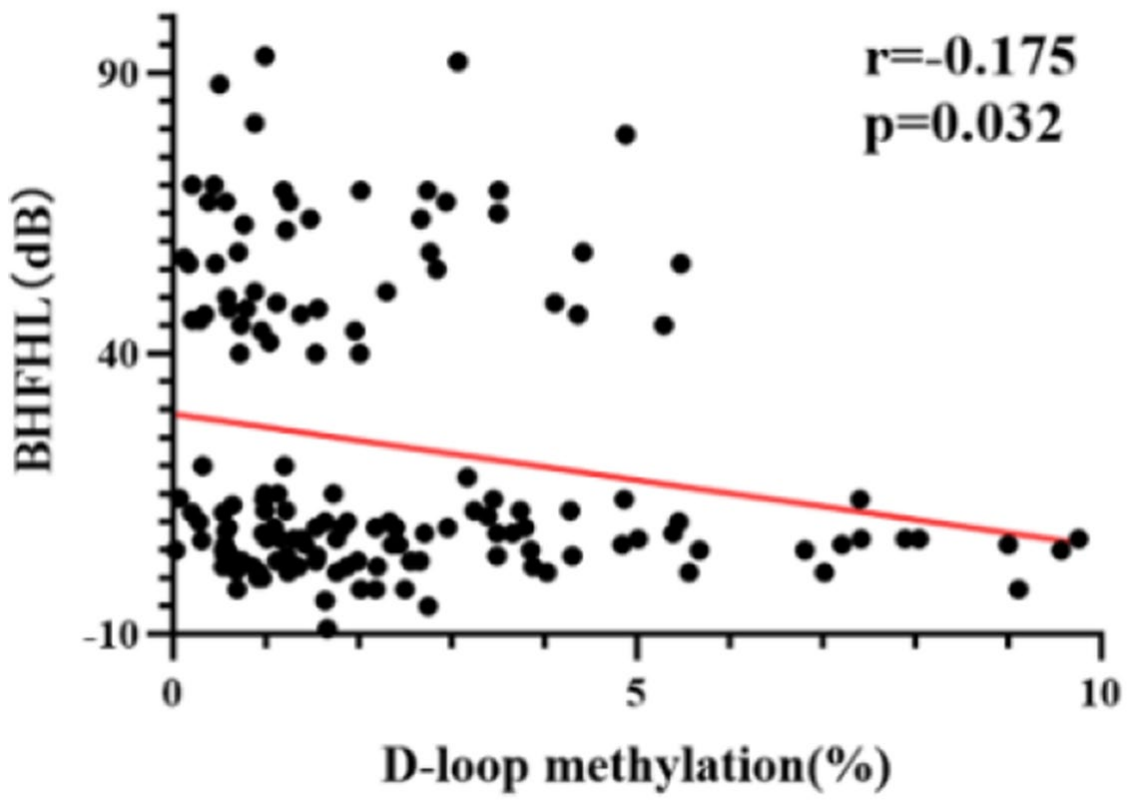
The correlation between DNA methylation levels and average hearing threshold of high-frequency bilateral ears in all subjects. The methylation level of the mtDNA D-loop region exhibits a statistically significant negative correlation with the average hearing threshold of high-frequency bilateral ears (r = −0.175, *p* < 0.05).

The methylation level of mtDNA D-loop region exhibits a statistically significant negative correlation with the average hearing threshold of high-frequency bilateral ears (r = −0.175, *p* < 0.05) (Figure 3). Specifically, a lower methylation level is associated with a higher average hearing threshold in high-frequency bilateral ears.

### 3.4 The correlation between DNA methylation levels and oxidative stress level in all subjects

**Figure 4:**
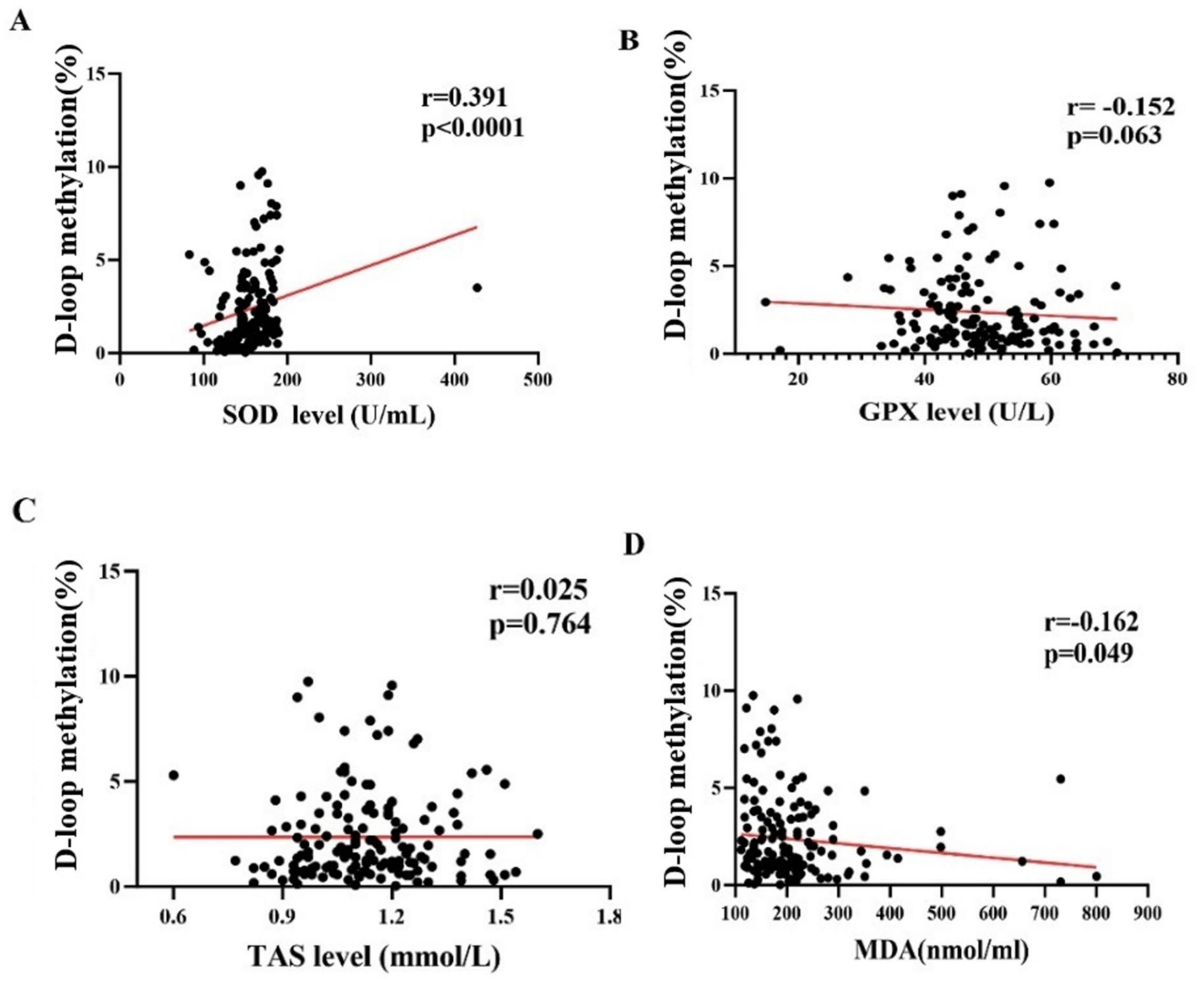
The correlation between DNA methylation levels and oxidative stress level in all subjects. **(A)** The methylation level of the mitochondrial DNA (mtDNA) D-loop region exhibits a statistically significant positive correlation with superoxide dismutase (SOD) (r = 0.391, *p* < 0.01). **(B)** The correlation between the methylation level of the mtDNA D-loop region and glutathione peroxidase (GPX) is weak (r = −0.152, *p* = 0.063) and not statistically significant. **(C)** The methylation level of the mtDNA D-loop region shows a positive correlation with total antioxidant status (TAS) (r = 0.025, *p* = 0.764), which is not statistically significant. **(D)** The methylation level of the mtDNA D-loop region is negatively correlated with malondialdehyde (MDA) (r = −0.162, *p* < 0.05), and this correlation is statistically significant.

The methylation level of the mtDNA D-loop region exhibits a statistically significant positive correlation with SOD (r = 0.391, *p <* 0.01) (Figure 4A). The correlation between the methylation level of the mtDNA D-loop region and GPX is weak (r = −0.152, *p =* 0.063) and not statistically significant (Figure 4B). The methylation level of the mtDNA D-loop region shows a positive correlation with TAS (r = 0.025, *p =* 0.764) (Figure 4C), which is not statistically significant. The methylation level of the mtDNA D-loop region is negatively correlated with MDA (r = −0.162, *p <* 0.05), and this correlation is statistically significant (Figure 4D).

### 3.5 Comparison of mtDNA-CN among three subject groups

**Figure 5:**
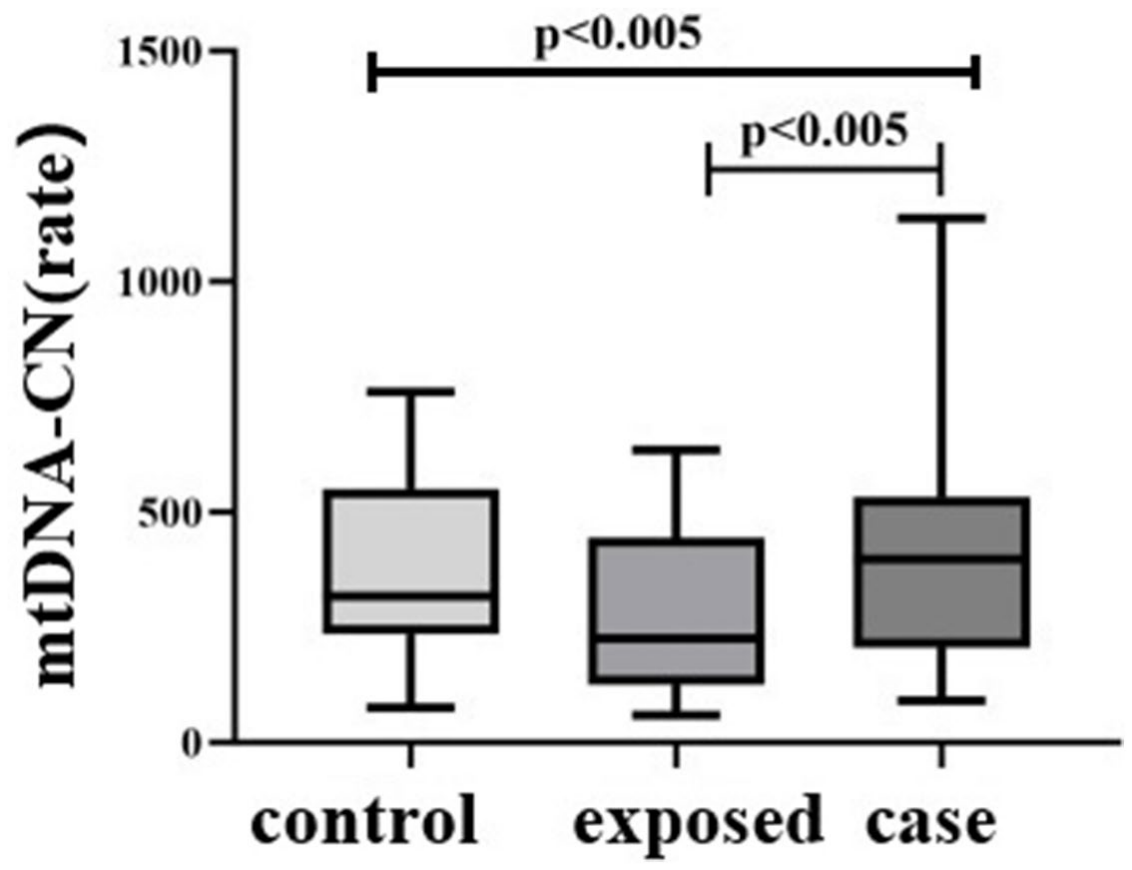
Comparison of mtDNA-CN among three subject groups. The differences in mtDNA-CN levels among the three groups were statistically significant (H = 9.213, *p* < 0.01), with the case group [397.7 (205.9, 532.1)] exhibiting higher levels compared to the blank control group [317.4 (234.6, 549.6)]. Additionally, the case group demonstrated significantly higher mtDNA-CN levels than the control group [225.1 (125.3, 445.0)], *p* < 0.05.

The differences in mtDNA-CN levels among the three groups were statistically significant (H = 9.213, *p <* 0.01), with the case group [397.7 (205.9, 532.1)] exhibiting higher levels compared to the control group [317.4 (234.6, 549.6)]. Additionally, the case group demonstrated significantly higher mtDNA-CN levels than the exposed group [225.1 (125.3, 445.0)], *p <* 0.05. Notably, the case group had the highest mtDNA-CN levels overall (Figure 5).

### 3.6 KEGG pathway analysis

**Figure 6:**
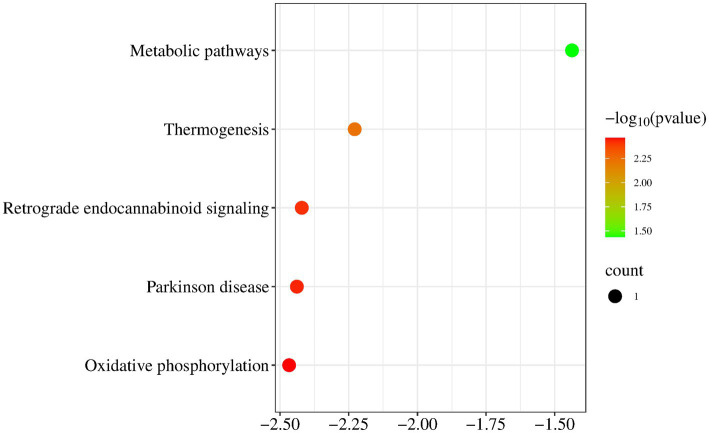
KEGG pathway analysis. KEGG pathway analysis of mitochondrial genes revealed a significant correlation between the MT gene and Metabolic pathways and Thermogenesis and Retrograde endocannabinoid signaling and Parkinson disease and Oxidative phosphorylation.

KEGG pathway analysis of mitochondrial genes (MT-ND1 and D-loop) revealed a significant correlation between the MT gene and Metabolic pathways and Thermogenesis and Retrograde endocannabinoid signaling and Parkinson disease and Oxidative phosphorylation (Figure 6).

### 3.7 Correlation between mtDNA-CN levels and DNA methylation level

**Figure 7:**
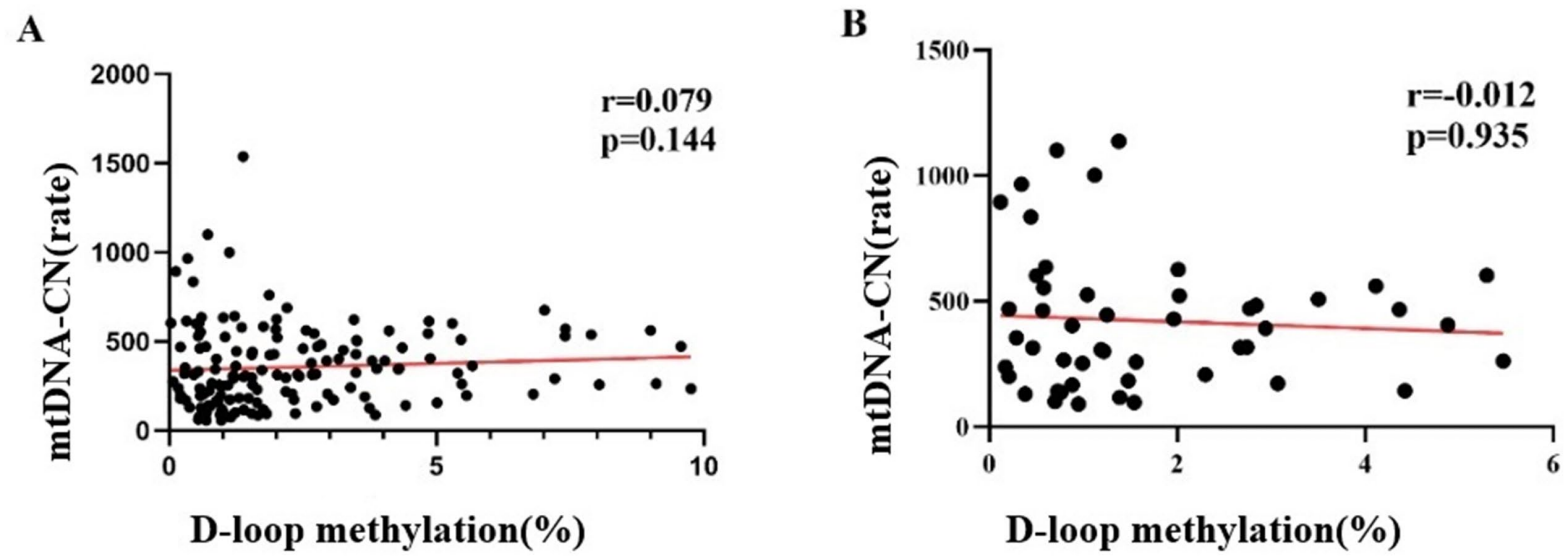
Correlation between mtDNA-CN levels and DNA Methylation Level The methylation level of the mtDNA D-loop region across the three study groups exhibited a positive correlation with mtDNA copy number; however, this association did not reach statistical significance (r = 0.079, *p* = 0.144) **(A)**. Within the case group, the methylation level of the mtDNA D-loop region demonstrated a negative correlation with mtDNA copy number. Specifically, a decrease in methylation level corresponded with an increase in copy number, yet this relationship was not statistically significant (r = −0.012, *p* = 0.935) **(B)**.

The methylation level of the mtDNA D-loop region across the three study groups exhibited a positive correlation trend with mtDNA -CN; however, this association did not reach statistical significance (r = 0.079, *p =* 0.144) (Figure 7A). Within the case group, the methylation level of the mtDNA D-loop region demonstrated a negative correlation trend with mtDNA -CN. Specifically, a decrease in methylation level corresponded with an increase in copy number, yet this relationship was not statistically significant (r = −0.012, *p =* 0.935) (Figure 7B).

### 3.8 Mediation effect

**Figure 8:**
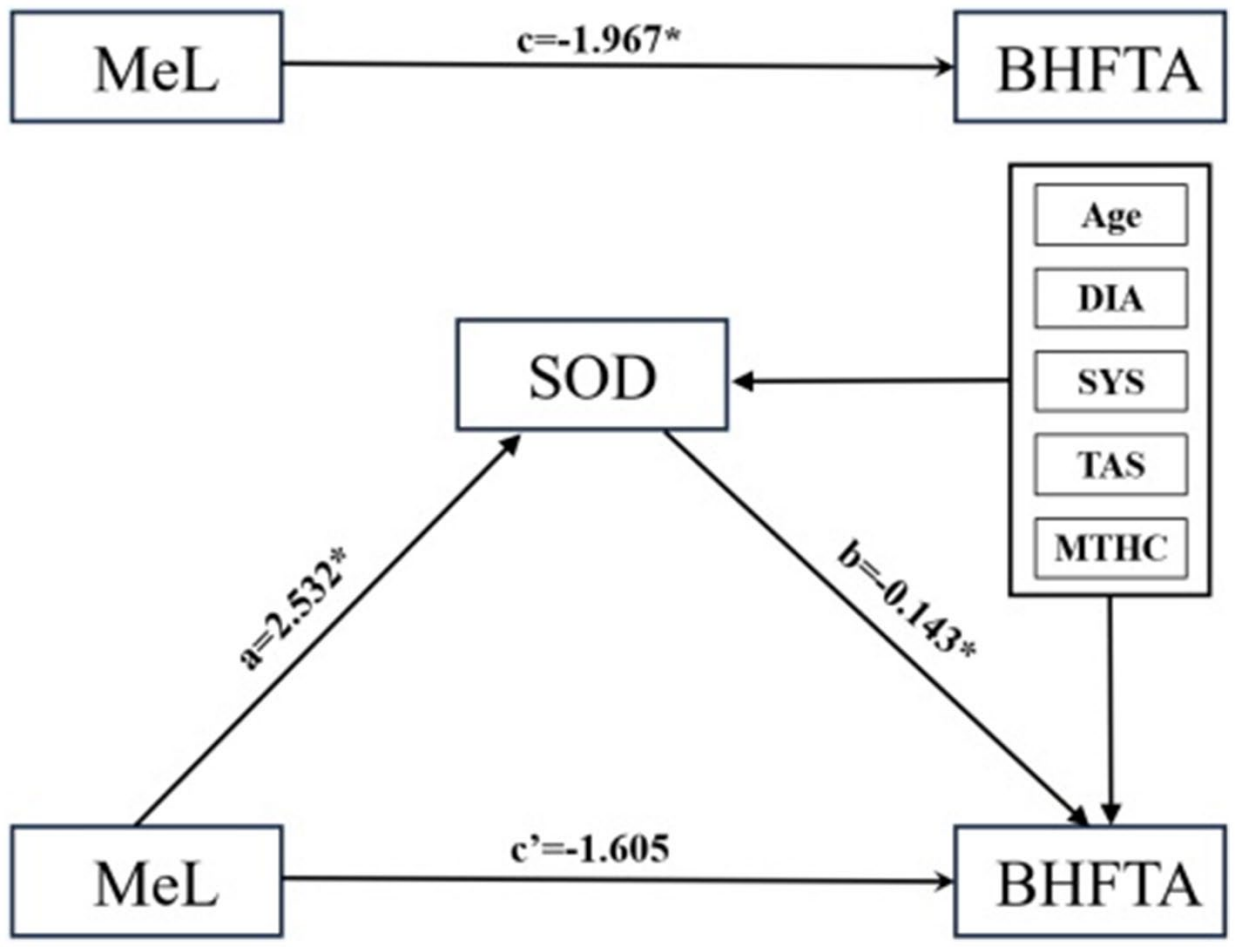
Mediating effect model. X, Y, M are the path coefficients between the control variables. Introduce SOD as the mediator variable, with age, diastolic pressure, systolic pressure, TAS, and mitochondrial copy number as control variables, and substitute them into the structural equation model. a represents the effect value of MeL on SOD, b represents the effect value of SOD on BHFTA, a*b represents the mediating effect, c’ represents the direct effect, and c represents the total effect.

In the mediating effect model, SOD fully mediates the effect of methylation on bilateral high-frequency hearing thresholds, indicating that the effect of methylation on bilateral high-frequency hearing thresholds may not be direct, but partially or entirely realized through SOD (Table 3, Figure 8).

**Table 3:** Overall effect, direct effect, and mediation effect decomposition table.

Variable	Effect value	Boot SE	*p*	95% BootCI	Effect size
a (MeL- SOD)	2.532*	1.202	0.037	0.176 ~ 4.887	
b (SOD - BHFTA)	−0.143*	0.064	0.028	−0.269 ~ −0.017	
c (MeL- BHFTA)	−1.967*	0.935	0.037	−3.800 ~ −0.134	
c’ (MeL-SOD-BHFTA)	−1.605	0.937	0.089	−3.441 ~ 0.231	81.596%
a*b	−0.362	0.048	0.000	−0.156 ~ 0.017	18.404%

a*b is the mediating effect, c’ is the direct effect, and c is the total effect. a and b are significant, while c’ is not significant. Although the 95% interval includes 0 in this case, it is still a complete mediating effect. The direct effect (−1.605) and the mediating effect (−0.362) account for 81.596 and 18.404% of the total effect (−1.967) respectively.

## 4 Discussion

Noise exposure triggers cochlear oxidative stress, a key driver of NIHL, by disrupting the balance between reactive oxygen species (ROS) production and antioxidant defense (Yamane et al., 1995; Juan et al., 2021). In our study, the NIHL case group exhibited elevated MDA (a marker of lipid peroxidation) alongside reduced SOD, GPX, and total antioxidant status (TAS), reflecting heightened oxidative damage and impaired antioxidant capacity. This aligns with animal studies showing noise-induced ROS surges in cochlear tissues and perilymph (Bagheri Hosseinabadi et al., 2019; Reastuty and Haryuna, 2021), and diminished SOD/GPX activity in noise-exposed rodents.

Consistent with these findings, diminished SOD expression has been reported in the brains of noise-exposed rats (Reastuty and Haryuna, 2021).

Superoxide dismutase 2 (SOD2), an isoform localized specifically within the mitochondrial matrix, plays a critical role in neutralizing mitochondrial superoxide. Studies demonstrate that SOD2 modulates the PI3K/MAPK signaling pathway, mitigating noise-induced hearing loss and kanamycin-induced mitochondrial DNA depletion in mice with a 4,834 gene mutation (Li et al., 2020). Additionally, reduced GPX activity has been documented following noise exposure (Samara et al., 2024). For example, noise exposure in rats induced vascular plexus swelling and significantly decreased GPX1 immunofluorescence intensity per unit area (Kil et al., 2007). These findings align with evidence showing that genetic deletion of GPX1 increases susceptibility to noise-induced cochlear damage and hearing loss (Ohlemiller et al., 2000). In the present study, serum TAS levels were lower in the case group than in controls. This observation is consistent with a study by Havlioglu et al. (2022) which compared 100 textile factory workers exposed to noise with 56 non-exposed healthy volunteers and found significantly reduced TAS levels in the noise-exposed group.

Unlike animal studies focusing on local (cochlear) oxidative stress, we show systemic perturbations (via serum markers), supporting Havlioglu et al. (2022) conclusion that noise-induced oxidative stress is not confined to the auditory system. This suggests that NIHL may be part of a broader systemic imbalance, a perspective underemphasized in prior work (Havlioglu et al., 2022).

The inverse relationship between SOD and MDA in our cohort directly links antioxidant depletion to oxidative damage, reinforcing SOD’s role as a critical gatekeeper—a mechanism supported by Li et al., who showed that SOD2 mitigates noise-induced hearing loss in mice, but here contextualized in a human occupational setting (Li et al., 2020).

In this study, it was observed that the methylation level of the mtDNA D-loop region was reduced in the case group. This reduction may be attributed to noise exposure, which induces the production of ROS and oxidative stress within the cochlea. The mtDNA D-loop region is particularly vulnerable to oxidative damage, potentially leading to diminished gene expression levels. The observed decrease in methylation of the mtDNA D-loop region may facilitate a compensatory increase in gene expression within this region, serving as a countermeasure to mitigate the oxidative damage. Research has demonstrated that the inhibition of DNA methyltransferase activity using the non-nucleoside specific inhibitor RG108, or the silencing of DNA methyltransferase-1 via siRNA, can significantly mitigate noise-induced elevations in auditory brainstem response (ABR) thresholds, hair cell damage, and auditory synapse loss (Zheng et al., 2021).

Additionally, other studies have suggested that elevated levels of lead (Pb) and cadmium (Cd) in children residing in electronic waste areas are associated with a slight negative trend in the methylation of the Rb1 and CASP8 promoters, whereas the methylation of the MeCP2 promoter exhibits a strong positive trend (Xu et al., 2020).

Similarly, global DNA methylation levels in patients with otosclerosis (OTSC) are reduced by 4.53-fold (females) and 4.83-fold (males) compared to healthy individuals (Bouzid et al., 2022). In contrast to prior studies that have not linked mtDNA methylation to oxidative stress, our data demonstrate that D-loop methylation correlates positively with SOD and negatively with MDA. This suggests that noise-induced ROS may directly disrupt methyltransferase activity or damage the oxidation-vulnerable D-loop region, thereby reducing methylation. Collectively, these findings suggest that noise exposure may perturb methylation patterns, contributing to its pathogenic effects.

In a study of noise-exposed male workers in China, it was observed that for every 1 dB (A) increase in annual cumulative noise exposure (CNE), the relative mtDNA-CN decreased by 0.014 units (Yang et al., 2024).

In animal models of hearing loss caused by diverse etiological factors, experimental results demonstrated a significant increase in mtDNA damage levels alongside reduced mtDNA-CN and diminished expression of PGC-1α and PGC-1β in aged mice (Oh et al., 2020). Epidemiological studies further revealed that among 300 infants with hearing loss and 200 healthy controls, individuals carrying mitochondrial gene mutations (A3243G, T5655C, and A14692G) exhibited lower mtDNA-CN compared to controls (Tang et al., 2019). The results of the above-mentioned literature reported lower mtDNA-CN in infants with hearing loss (vs. controls), contrasting with our finding of elevated mtDNA-CN in NIHL cases. We propose that this difference reflects a compensatory mechanism: as noise-induced D-loop hypomethylation disrupts mtDNA regulation, cells upregulate replication to maintain mitochondrial function—a hypothesis supported by Coppede and Stoccoro (Coppede, 2024), who noted inverse correlations between D-loop methylation and mtDNA-CN. Methylation levels of the mtDNA D-loop region are positively correlated with SOD and negatively correlated with MDA. Noise exposure may disrupt the balance between antioxidant and pro-oxidant enzymes in hair cells, leading to ROS overproduction and oxidative stress in cochlear cells (Gu et al., 2021).

The mtDNA D-loop region is particularly susceptible to oxidative damage, which may reduce gene expression levels and decrease methylation. Mutations in this region can impair mtDNA replication fidelity, triggering compensatory increases in mtDNA-CN. Studies suggest that inhibiting DNA methylation via the LRP1-PI3K/AKT pathway reduces oxidative stress-induced mitochondrial apoptosis, thereby alleviating cisplatin-induced hearing loss (He et al., 2022).

In age-related hearing loss models, the DNA methylation inhibitor 5-azacytidine reduces SOD2 methylation, mitigates oxidative stress, and inhibits H2O2-induced cell apoptosis (Li et al., 2020). Additionally, mtDNA D-loop methylation is dynamically regulated during the progression of neurodegenerative diseases. In this study, SOD mediated the full effect of methylation on bilateral high-frequency hearing thresholds, underscoring its critical role in auditory regulation. Methylation of the mtDNA D-loop region suppresses mtDNA replication, with methylation levels inversely correlating with mtDNA–CN (Coppede and Stoccoro, 2019).

Similar compensatory mechanisms have been observed in cerebral autosomal dominant arteriopathy with subcortical infarcts and leukoencephalopathy (CADASIL) patients, where mitochondrial dysfunction leads to reduced D-loop methylation and elevated mtDNA-CN (Zhang et al., 2022).

Among the three study groups, the case group exhibited the lowest levels of SOD, GPX, and TAS, along with the highest level of MDA. mtDNA-CN in the case group was the highest, while methylation levels in the D-loop region were the lowest. Additionally, the exposed group showed a higher absolute lymphocyte count and a lower PLR compared to both the control group and the case group. The absolute lymphocyte count is an important immune indicator in the human body, while PLR serves as a representative marker of inflammation levels. These findings suggest that noise exposure in the control group may have stimulated protective immune responses, reducing systemic inflammation and enhancing antioxidant capacity. This could explain the observed decrease in oxidative damage (lower MDA), reduced mtDNA-CN (due to compensatory regulation), and increased mtDNA D-loop methylation for the exposed group. Environmental factors such as noise exposure and oxidative stress are known to modulate DNA methylation patterns. Our study indicates that changes in methylation may affect genes involved in cellular protection. This study has several limitations. Participants were recruited from a single occupational center in Shenzhen, which may limit generalizability to other populations. The sample size (*n* = 150) also restricts power for detecting subtle effects or subgroup differences. Although age and sex were matched, unmeasured confounders—such as lifestyle factors, genetic background, and co-exposures to other occupational hazards—may affect oxidative stress and methylation measures. While SOD mediated the methylation–hearing threshold relationship, the precise mechanisms (e.g., SOD’s role in regulating DNA methylation) remain unexplored. Future research should investigate the following recommendations: implement longitudinal designs with repeated measures to establish temporal relationships between methylation changes and hearing loss; use genetic and pharmacological interventions (e.g., SOD2 knockout, DNMT modulators) in experimental animal models to clarify mechanistic pathways; improve control of confounders through detailed covariate collection and adjusted analyses; and evaluate the clinical utility of combined biomarkers (e.g., D-loop methylation, SOD, MDA) for risk prediction and early intervention.

## 5 Conclusion

This study investigated the relationships among occupational noise exposure, oxidative stress, mitochondrial DNA (mtDNA) D-loop methylation, mtDNA copy number (mtDNA-CN), and noise-induced hearing loss (NIHL) in a matched cohort of 150 occupational workers. Key findings indicate that NIHL cases exhibit elevated high-frequency hearing thresholds, reduced antioxidant capacity (lower SOD and TAS, higher MDA), D-loop hypomethylation, and a biphasic shift in mtDNA-CN—initially decreased in exposed controls but elevated in NIHL cases, suggesting compensatory mtDNA replication.

Notably, we identified a novel pathway in which SOD mediates the effect of D-loop methylation on high-frequency hearing thresholds, integrating oxidative stress with mitochondrial epigenetic regulation. This provides new mechanistic insight into NIHL pathogenesis, highlighting the role of mtDNA methylation not as a mere biomarker but as a functional element in noise-induced damage.

These findings advance the theoretical framework of NIHL by connecting oxidative stress with mitochondrial epigenetics and suggest potential clinical applications: D-loop methylation and SOD activity may serve as biomarkers for early detection and risk stratification, while mtDNA-CN dynamics could help monitor mitochondrial adaptation. Restoring SOD activity or modulating methylation may offer new strategies for preventing NIHL in high-risk populations.

In summary, this study elucidates a SOD-mediated mtDNA epigenetic mechanism in NIHL, providing a foundation for biomarker-guided interventions and further research into mitochondrial regulation in environmental hearing loss.

## Data Availability

The raw data supporting the conclusions of this article will be made available by the authors, without undue reservation.

## Footnotes

^1^ http://bioinfo.org/kobas/

## Glossary

NIHL: Noise-induced hearing loss
mtDNA D-loop region: mitochondrial DNA D-loop region
mtDNA-CN: mitochondrial DNA copy number
PCR: Polymerase Chain Reaction
SOD: Superoxide Dismutase
GPX: Glutathione Peroxidase
TAS: Total Antioxidant Status
MDA: malondialdehyde
KEGG: Kyoto Encyclopedia of Genes and Genomes
ROS: reactive oxygen species
RNS: reactive nitrogen species
AIF: apoptosis-inducing factor
EndoG: endonuclease G
ATP: adenosine triphosphate
OHCs: outer hair cells
CpG: Cytosine-phosphate-Guanine
nDNA: nuclear DNA
D-loop: displacement loop
mtDNA-CN: mitochondrial DNA copy number
qPCR: Quantitative Polymerase Chain Reaction
ANOVA: one-way analysis of variance
PLR: platelet-to-lymphocyte ratio
BHFHL: bilateral high-frequency hearing loss
SOD2: Superoxide dismutase 2
PI3K/MAPK: Phosphoinositide 3-kinase/Mitogen-Activated Protein Kinase
RG108: N-Phthalyl-L-tryptophan
SiRNA: small interfering RNA
ABR: auditory brainstem response
Pb: lead
Cd: cadmium
Rb1: Retinoblastoma 1 protein
CASP8: Caspase-8
MeCP2: Methyl CpG binding protein 2
OTSC: otosclerosis
CNE: cumulative noise exposure
PGC-1α: Peroxisome proliferator-activated receptor-gamma coactivator 1-alpha
PGC-1β: Peroxisome proliferator-activated receptor-gamma coactivator 1- Beta
LRP1-PI3K/AKT: Low-Density Lipoprotein Receptor-Related Protein 1 - Phosphoinositide 3-Kinase/Protein Kinase B
CADASIL: subcortical infarcts and leukoencephalopathy
